# CRISPRi*-*mediated suppression of *E. coli* Nissle 1917 virulence factors: A strategy for creating an engineered probiotic using *csgD* gene suppression

**DOI:** 10.3389/fnut.2022.938989

**Published:** 2022-08-01

**Authors:** Mohd W. Azam, Asad U. Khan

**Affiliations:** Medical Microbiology and Molecular Biology Lab, Interdisciplinary Biotechnology Unit, Aligarh Muslim University, Aligarh, India

**Keywords:** bacterial amyloids, curli, biofilm, CRISPRi, dCas9, *csgD*, *E. coli*, probiotic

## Abstract

**Background:**

Biofilm formation is a complex phenomenon, and it is the causative agent of several human infections. Bacterial amyloids are involved in biofilm formation leading to infection persistence. Due to antibiotic resistance, their treatment is a great challenge for physicians. Probiotics, especially *E. coli* Nissle 1917 (EcN), are used to treat human intestinal disorders and ulcerative colitis. It also expresses virulence factors associated with biofilm and amyloid formation. EcN produces biofilm equivalent to the pathogenic UPEC strains.

**Methods:**

CRISPRi was used to create the knockdown mutants of the *csgD* gene (*csgD*-KD). The qRT-PCR was performed to assess the expression of the *csgD* gene in *csgD*-KD cells. The *csgD*-KD cells were also evaluated for the expression of *csgA, csgB, fimA, fimH, ompR, luxS*, and *bolA* genes. The gene expression data obtained was further confirmed by spectroscopic, microscopic, and other assays to validate our study.

**Results:**

CRISPRi-mediated knockdown of *csgD* gene shows reduction in curli amyloid formation, biofilm formation, and suppression of genes (*csgA, csgB, fimA, fimH, ompR, bolA, and luxS*) involved in virulence factors production.

**Conclusion:**

Curli amyloid fibers and fimbriae fibers play a critical role in biofilm formation leading to pathogenicity. CsgD protein is the master regulator of curli synthesis in *E. coli*. Hence, curli amyloid inhibition through the *csgD* gene may be used to improve the EcN and different probiotic strains by suppressing virulence factors.

## Introduction

Bacterial cells may survive as aggregates of cells or in planktonic form. The former state is called biofilm. Biofilms are heterogeneous communities of microbial cell populations residing in an extracellular matrix environment secreted by themselves. They tend to colonize a range of surfaces that may be biotic or abiotic in nature (1). Bacterial cells in the biofilm stage are advantageous over the planktonic form in several aspects: cells in biofilms require higher concentrations of antibiotics or antimicrobial agents due to the reduced penetrability of the antimicrobial agents, increased tolerance to disinfectants, enhanced bacterial conjugation capacity, increased interspecific metabolic cooperation, increased cell-cell communication, that is, quorum-sensing, and increased tolerance to host immune system, that is, components of the innate and adaptive inflammatory defense system of the body (1, 2).

*Escherichia coli* belongs to one of the most genetically as well as phenotypically diverse bacterial species. It can exist in the mammalian digestive tract as a harmless commensal and as a pathogen causing significant morbidity and mortality throughout the world. The biofilm-forming ability of *E. coli* is widely studied from the nonpathogenic strain *E. coli* K12 strains. Pathogenesis involving *E. coli* biofilm is well-studied in diarrheagenic *E. coli*, enteroaggregative *E. coli*, enterotoxigenic *E. coli* (ETEC), and adherent-invasive *E. coli*. The latter is connected to Crohn's disease (CD) origin and perpetuation, a type of inflammatory bowel disease. Biofilm production by extraintestinal pathogenic *E. coli* (ExPEC) was mainly reported in uropathogenic *E. coli* (UPEC) (2–4). UPEC causes 80% to 90% of community-acquired urinary tract infections (UTIs), 30–50% of nosocomial UTIs, and most recurrent UTIs (RUTIs) (5). Another notable example of *E. coli* pathogenicity is a hemolytic uremic syndrome (HUS) caused by *E. coli* O157:H7 or other serotypes of Shiga toxin-producing *E. coli* (STEC) such as *E. coli* O104:H4 (6).

Probiotics are defined as live microorganisms beneficial to the host organism, regarding their health, provided they are administered in adequate amounts (7). Probiotics attach themselves to intestinal epithelial tissue. It enhances the epithelial barrier function, which is critical to the host defense system for the protection against infection and inflammations caused due to pathogens. Probiotic bacteria like *E. coli* Nissle 1917 (EcN), *Lactobacillus* species, *Streptococcus* species, and *Bifidobacterium* species work to boost the immune system either individually or in combination with other probiotic strains. Probiotics biofilm presence in a healthy gut promotes nutrient exchange between the host and microbiota, and it increases the persistence of bacterial cells. Probiotic bacteria can permanently colonize the mucosa of the host through biofilm formation that prevents the colonization of the pathogenic microbes. Acting as a barrier, the immune system of mucosa prevents pathogenic microbes and other immunogenic agents from passing through the mucosa into the host (7, 8). *EcN* is reported to suppress the biofilm formation of pathogens including enterohemorrhagic *E. coli* (EHEC), *Pseudomonas aeruginosa, Staphylococcus aureus*, and *S. epidermidis*. Moreover, EcN suppressed the dual-species EHEC biofilm by 14-fold as compared to its single-species biofilms. Whereas, this figure is found to be 1,100-fold for *S. aureus* and 8,300-fold for *S. epidermidis*, in contrary to the commensal *E. coli* which does not inhibit bacterial biofilms. It was observed that EcN secretes DegP, a bifunctional (protease and chaperone) periplasmic protein, outside the cells and regulates other biofilms (8).

EcN strain has been used to attenuate intestinal disorders like inflammatory bowel disease and ulcerative colitis in human beings and veterinary clinical treatments. EcN, along with other intestinal microorganisms, including probiotic, non-probiotic, and pathogenic bacteria, colonizes the intestinal surface and stimulates epithelial cells for the production of human β-defensin 2. This colonization causes enhancement in the immune response of the host and stimulates the secretion of microcins. The microcins function as antimicrobials agents for specific strains and inhibit the growth and biofilm formation in other strains of *E. coli* (7).

Curli fibers are coiled, rigid, 2- to 5-nm thick, and entangled into a dense cluster surrounding the bacterial cell. These fibers are synthesized through a nucleation-dependent self-assembly process, and they acquire an amyloid-fold as their native structure/conformation. Curli fibers are synthesized through distinct secretion assembly pathways called nucleation-precipitation pathway or through the type VIII secretion system (T8SS) (9). Amyloid fibers are unique biopolymers comprised of protein subunits, and they are connected through non-covalent interactions (10). In bacteria, curli fibers form the amyloid fibers and are expressed on cell surfaces of the enteric bacteria (mostly) (11). Curli amyloid fibers are rich in β-sheet structure, and these β-sheet strands are arranged perpendicular to the fiber axis. This arrangement is called the “cross beta structure”, and it is the characteristic feature of amyloid proteins, including most of the human amyloid diseases associated proteins. The residues in the core of the fiber contribute to amyloid stabilization which provides tensile strength to the fibers related to steel and other inorganic materials. These properties of amyloid fibers have created a challenge, and their accumulation causes many diseases, especially neurodegeneration. Several studies have reported that oligomeric intermediates are formed during the process of amyloidogenesis and are mainly cytotoxic. Many organisms have evolved mechanisms to utilize this amyloid-fold means that these amyloids can also take part in physiological functions, and they are called functional amyloids. These “*functional amyloids*” exist in all domains of life and are involved in a range of processes, including cell signaling, storage, structural scaffolding, and even in-memory persistence. Several bacterial species utilize curli amyloid fibers as a significant component of their extracellular biofilm matrix. They have evolved distinct types of cell machinery to coordinate the secretion of amyloid fibers (10–12).

The curli system of *E. coli* is comprised of seven curli-specific genes (*csgA-G*), which are clustered into two divergently transcribed operons, namely *csgBAC* and *csgDEFG*. The *csgA* and *csgB* encode the structural components of the curli fiber (CsgA and CsgB), *csgC* encodes a periplasmic chaperone (CsgC), *csgE*, c*sgF*, and *csgG* encode the secretion-assembly machinery (CsgE, CsgF, and CsgG), and the product *csgD* gene CsgD acts as a transcriptional activator of the *csgBAC* operon (9). *In E. coli*, the functional amyloid fiber (curli fiber) of the extracellular matrix are heteropolymers of CsgA and CsgB proteins. The CsgA and CsgB are natively disordered or intrinsically disordered proteins (IDPs) having flexible and aggregation-prone regions. With the assistance of proteins encoded on the *csgDEFG* operon, the *csgA* and *csgB* genes are chaperoned and secreted to the surface of the cell (13). CsgD is a transcription factor belonging to FixJ/LuxR/UhpA family and is a master regulator of biofilm (14, 15). *CsgD* transcriptionally activates *csgBAC and csgDEFG* operons for curli production. Besides, CsgD is involved in several cellular activities like it regulates several genes critical to biofilm formation, for example, cellulose biosynthesis genes, cell-surface structures, and stress response functions (12, 15). Moreover, CsgD obstructs the flagella formation by inhibiting the transcription of the master regulator of flagella formation *FlhDC* target genes: *fliA, fliE, fliFGHIJK*, and *flgM*, which are required for the planktonic growth of cells (14). CsgD expression is controlled at both transcriptional and translational levels by many regulatory proteins. Many transcription factors have been identified which play a role in *csgD* promoter regulation including MqsA, BasR, MlrA, CpxR, Crl, CRP, CsgD, IHF, RstA, OmpR, RcdA, Cra, and H-NS (13, 14). Several parameters like temperature, amount of oxygen, osmolarity, cell density, and starvation also play an important role in *csgD* gene expression, which indirectly affects *csgBAC* operon expression (14). Curli amyloid protein fibers form an important constituent of the biofilm matrix (ECM) of UPEC. These amyloid fibers induce the immunogenic complexes formation with DNA to stimulate the autoimmune responses. They are also highly proinflammatory and can neutralize cathelicidin (LL-37), a soluble human antimicrobial peptide that protects from UTIs (15).

Curli amyloids are associated with many human diseases such as Parkinson's disease and Alzheimer's disease involving amyloid curli (*E. coli*) and FapC (*Pseudomonas*), Reactive Arthritis (ReA) involving curli amyloid (*S. Typhimurium*), and Systemic lupus erythematosus (SLE) involving curli amyloid and other bacterial amyloids (*S. Typhimurium, E. coli*) (16). The gut microbiota is engaged in human physiology through various processes, and its irregularities are linked to many diseases, including Alzheimer's disease. The gut microbiota appears to have a role in Alzheimer's disease development by affecting neuroinflammation and metabolic balance (17). Another study involving the human amyloid α-synuclein (αSyn) overexpressing mice shows that colonization with curli-producing *E. coli* promotes αSyn pathology in the gut and the brain. Curli expression is essential for *E. coli* to aggravate αSyn-induced behavioral abnormalities, such as intestinal and motor dysfunction (18).

The genome sequence of *E. coli* Nissle suggested that it is closely related to the CFT073 strain of UPEC (19). Nissle 1917 possesses several genes-producing virulence factors. It includes Ag43 and fimbriae such as F1C fimbriae, type 1 fimbriae. EcN is a strong producer of cellulose and curli fibers and has several proteases and an array of iron acquisition systems. Curli fibers, F1C fimbriae, type 1 fimbriae, Ag43, and cellulose play an essential role in biofilm formation (20).

The central hypothesis of our study was to design the probiotic *E. coli* Nissle 1917 (EcN) strain with minimized curli amyloid and other virulence factors productions by editing the *csgD* gene using CRISPR interference (CRISPRi). CRISPRi utilizes dCas9 protein which is a catalytically inactive version of Cas9 protein and lacks endonucleolytic activity. Both nuclease domains of Cas9 protein, RuvC-like (D10A) and HNH (H840A), were mutated by creating point mutations. This mutant Cas9 (dCas9) will be unable to make double-stranded DNA cuts upon binding to the target site (21).

Since the *csgD* gene is involved in amyloid formation and controls several biofilm-forming genes. EcN strain is known to produce several types of virulence factors that may have pathogenic effects on human beings. CRISPRi gene-editing system was used to inhibit the *csgD* gene expression. Several genes have essential roles in biofilm formation, such as *ompR (*involved in two-component systems, EnvZ-OmpR*)*, curli genes *(csgA and csgB)*, fimbriae genes *(fimH and fimA)*, and morphogene *bolA* and *luxS* gene of quorum sensing, were considered for gene expression analysis in CRISPRi-mediated *csgD*-KD cells. By knocking down the *csgD* gene, the genes mentioned above were also suppressed in *csgD-*KD cells, making EcN strains less virulent or avirulent for probiotic purposes.

## Materials and methods

### Bacterial strains and plasmids

*E. coli* Nissle 1917 (EcN) strain and *E. coli* K12 strain were used for this study. Two commercially purchased plasmid vectors from the Addgene repository, USA, were used: pdCas9 plasmid (Addgene no. 44249) for the expression of dCas9 endonuclease and pgRNA or sequence-specific sgRNAs expression (Addgene no. 44251).

### Sequence-specific sgRNA cloning

Two sets of primers were designed to generate the sequence-specific sgRNAs (Supplementary Table S1). The complementary sequences to the *csgD* gene (20 bp sgRNA sequences adjacent to PAM region, i.e., immediately following 5'-CCN-3') along with 35 nucleotide parts of the dCas9 handle were commercially synthesized in the form of primers. Inverse PCR reactions were performed to insert the 20 bp target sgRNA sequences in pgRNA plasmid, as per the protocol used by Larson et al. (20). Before performing PCR, both forward and reverse primers were phosphorylated. The PCR results were verified by running a 1% agarose gel. The correct bands (~2.5 kb) were eluted by using a gel extraction kit (Promega), and the eluted sequences were freed from template strands by *Dpn*I enzyme digestion and ligated by a quick, blunt end ligation kit (New England Biolabs). The ligated products were transformed into chemically competent *E. coli* top 10 cells through the heat shock method. Colony PCR was performed to identify the correct clones (Supplementary Figure S1). The desired colony PCR bands on agarose gel were eluted and sent for DNA sequencing with the forward primer. The sequencing confirmed plasmids having the correct sgRNA sequence were named SgcsgA and SgcsgB. Cells with SgcsgA plasmid were referred to as KD 'mutant A', while the cells with SgcsgB plasmid were referred to as KD 'mutant B'.

The sgRNA plasmid was isolated from clones confirmed from sequencing (SgcsgA and SgcsgB). The confirmed sgRNA plasmid was co-transformed along with the pdCas9 plasmid in chemically competent *E. coli* Nissle (EcN) strain and *E. coli* K12 strain by heat shock method. EcN cells having SgcsgA plasmid were named TEA and EcN cells with SgcsgB were called TEB. Similarly, K12 cells with SgcsgA plasmid were named TKA and K12 cells with SgcsgB plasmid were named TKB. The single colonies of each *csgD*-KD cell were picked up and cultured in LB broth along with ampicillin and chloramphenicol for further experiments (Supplementary Figure S2).

### Biofilm-related gene expression assessment in *csgD* knockdown cells

The gene expression was assessed by using qRT-PCR. The total RNA isolation was done by the TRIzol method as done previously (21). About 1% of the overnight culture grown at 37°C was inoculated in a fresh autoclaved LB media supplemented with ampicillin 100 μg/mL, chloramphenicol 25 μg /mL, and inducer anhydrotetracycline 2 μM (hydrochloride, aTc) and grown on shaking incubated at 37°C until the OD reaches 0.4. Control cells have pdCas9 and sgRNA plasmids, and control media is supplemented with only antibiotics, no inducer.

The isolated total RNA was treated with RNase-free DNase to remove DNA impurities. The cDNA was prepared by the cDNA Reverse Transcription Kits (Applied Biosystems, USA). SYBR Green PCR master mix (ThermoFisher) was used for RT-PCR along with 150 ng of cDNA sample, and forward and reverse RT primers. The 16S rRNA was taken as endogenous control, and PCR conditions were 95°C for 10 min, 95°C for 15 s, 60°C for the 30 s, and finally 72°C for 30 s.

### Crystal violet assay

Overnight culture of co-transformed cells grown in LB broth supplemented with antibiotics and inducer aTc (2 μM) was diluted 1:200 in fresh sterile LB media supplemented with ampicillin, chloramphenicol, and aTc. The biofilm was grown in a “U” shaped 96-well microtiter plate for the CV assay as previously done by Zuberi et al. (22). The 100 μL of the diluted overnight culture was dispensed in each well of a microtiter plate and was kept for 48 h at 26°C without shaking. After incubation, the planktonic cells were removed, and the wells were washed with 1X PBS (pH 7.4). About 37% formalin and 2% sodium acetate were used to fix the biofilm, and plates were incubated at 4°C for 4 h. After incubation, the staining was done by dispensing 150 μL of crystal violet (CV) solution (0.1%) in each well, and the plate was incubated for 15 to 20 min at room temperature. After this step, the wells were washed with PBS, and the bound dye was dissolved in 95% absolute alcohol (100 μL in each well). After 5 min of incubation at room temperature, the microtiter plate was read at 630 nm using a BIORAD microtiter plate reader.

### Thioflavin and Congo red fluorescence assay

The biofilm was grown in 96-well plates as described in the CV assay performed in the earlier study (23). After the 48 h growth of biofilm, the media having planktonic cells was discarded, and biofilm was dissolved in filtered autoclaved ddH2O. A 250-μL ddH2O was used for each well of biofilm. These dissolved biofilm cells were used for further ThT and Congo red assays. About 10 μg/mL concentration of the ThT was used, and the sample was kept in the dark for 2 h at room temperature, and fluorescence spectra were recorded at 450 nm excitation wavelength. Congo red samples were added with 10 μg/mL Congo red and incubated at 37°C for 3 h in the dark. The Congo red spectra were recorded at 540 nm excitation wavelength.

### Cell viability assay (XTT reduction assay)

Biofilm was grown similar to the CV assay, and XTT reduction/cell viability assay was done as reported earlier by Zuberi et al. (23). Filter sterilized menadione solution in acetone (0.4 mM) and 1 mg/mL XTT solution in PBS was prepared. Media was removed from wells to remove the planktonic cells, and biofilm was washed with PBS. A fresh mixture of XTT and menadione in 20:1 by volume was prepared. About 42 μL of XTT and 158 μL of PBS per well were dispensed and incubated in the dark for 3 h at 37°C. After incubation, the change in color intensity was read at 490 nm with the help of a microtiter plate reader.

### Congo red agar plate assay for curli production

LB agar plates with Congo red (30 μg/mL) supplemented with 100 μg/mL ampicillin, 25 μg/mL chloramphenicol, and aTc inducer (2 μM) were prepared, as done earlier (24). The control plates were prepared similarly except that it has no inducer. The plate was streaked with an overnight culture of knockdown cells and was incubated for 48 h at 26°C.

### Confocal laser scanning microscopy

Control and knockdown cells biofilm were grown in confocal dishes (Coverglass-Bottom *Dish*, genetix) at 26°C for 48 h. The media having planktonic cells were removed and biofilm was washed with PBS. The biofilm was stained with 0.2 μg/mL concentration DAPI (4′, 6-Diamidine-2′-phenylindole dihydrochloride, Sigma) dye and incubated for 1 h at room temperature. The pictures were captured under the Zeiss LSM 780 confocal laser scanning microscope (CLSM, Germany) at a 20-μm scale.

### Transmission electron microscopy

Biofilm was grown as described in the CV assay, and TEM was performed as reported earlier (24). After 48 h of growth of biofilm, the planktonic cells were removed, and biofilm was washed with filtered autoclaved ddH_2_O. After washing, the biofilm was dissolved in filtered autoclaved ddH_2_O. The cells were observed under the TEM (JEM 2100, Jeol, Tokyo, Japan) at a 500-nm scale.

### Statistical analysis

All the experiments reported in this article were performed in triplicates. The results were compared with control for the analysis of student *t*-test, two-tailed hypothesis (^*^P < 0.05, *t*-test, two-sided; ^**^P < 0.005, *t*-test, two-sided), and one-way analysis of variance (ANOVA). ANOVA was done from the website http://www.physics.csbsju.edu/stats/anova/html. The *p-*values between *p* < 0.05 and *p* < 0.001 were considered statistically significant.

## Results and discussion

### Downregulation of *csgD* gene of EcN and K12 strain

**Figure 1:**
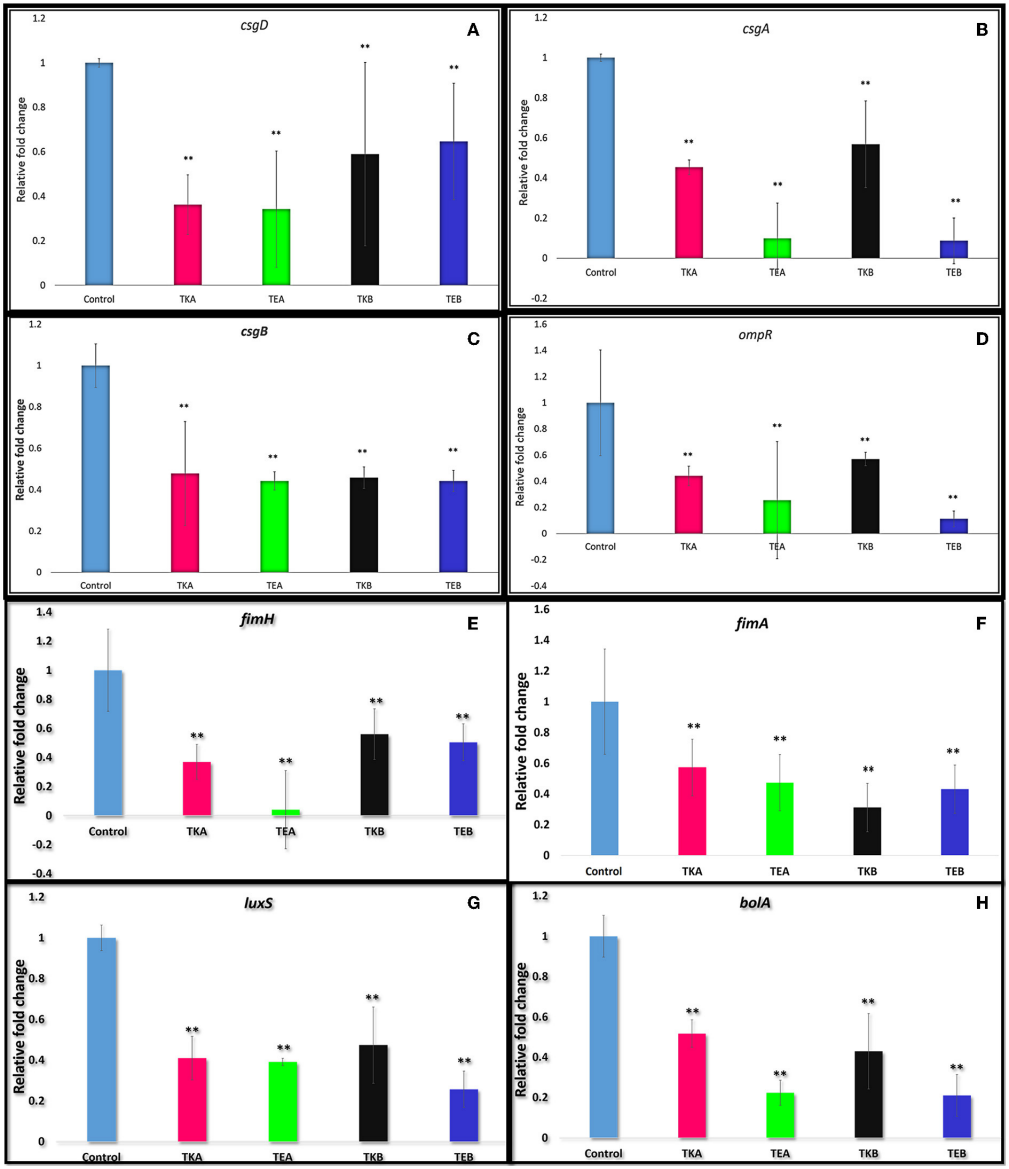
(**A)** *csgD* gene downregulation quantified by quantitative real-time PCR in *E. coli* Nissle 1917 (EcN) and K12 strain. The *csgD*-KD mutant A showed a 65.8% suppression in EcN (TEA) and in K12 cells and 63.8% reduction (TKA) as compared to the control cells (cells having no aTc induction but have both plasmids). The *csgD*-KD mutant B showed a 35.4% reduction in EcN (TEB) and 41.0% reduction in K12 (TKB). **(B–H)** Downregulation of the different genes in *csgD*-KD cells of EcN (TEA and TEB) and K12 (TKA and TKB). **(B)** Downregulation of *csgA* gene. **(C)** Downregulation of *csgB* gene. **(D)** Downregulation of *ompR* gene. **(E)** Downregulation of *fimH* gene. **(F)** Downregulation of *fimA* gene. **(G)** Downregulation of *luxS* gene. **(H)** Downregulation of *bolA* gene. The gene expression data was represented in fold change. One-way ANOVA analysis was done, and the *p-value < 0.05,**p-value < 0.005 was considered statistically significant. The 16s rRNA gene was used as endogenous control.

Th*e csgD* gene was knockdown by using the CRISPRi tool using pgRNA and pdCas9 plasmids, and the expression level was checked by performing quantitative real-time PCR (qRT-PCR). Two KD mutants A and B were targeted at different positions in the *csgD* gene. The relative fold change in gene expression was calculated from the RQ value. The relative fold change of control was 1 ± 0.0189 and of TKA was 0.3625 ± 0.1340, that is, 63.8% gene suppression was observed. The KD mutant (*csgD*-KD) A showed a 65.8% suppression in EcN (TEA), and in K12 cells, 63.8% suppression (TKA) was observed as compared to the control cells (cells having no aTc induction). The KD mutant (*csgD*-KD) B showed a 35.4% gene suppression in EcN (TEB) and 41% in K12 (TKB) (Figure 1A).

### Downregulation of biofilm-forming genes in *csgD*-KD cells of EcN and K12

As we already know, the *csgD* gene is involved in the curli synthesis and biofilm formation, so we have also assessed the expression level of a few genes essential in biofilm formation (Figures 1B–H) in the same *csgD-*KD cells. The reduction in the gene expression level of *csgD-*KD EcN and K12 cells was compared with control cells, and their respective percent gene suppression is shown in Table 1.

**Table 1:** Gene suppression level of different genes critical to the biofilm formation in *E. coli* in *csgD-KD* cells of *E. coli* Nissle 1917 and K12 strains (Note that TKA = K12 cell with mutation A, TEA = EcN cell with mutation A, TKB = K12 cell with mutation B, TKB = EcN cell with mutation B).

**Genes**	**TKA**	**TEA**	**TKB**	**TEB**
*csgA* (Figure 1B)	54.6	90.1	43.2	91.4
*csgB* (Figure 1C)	52.2	55.8	54.2	56.8
*fimA* (Figure 1F)	42.7	52.6	68.8	56.8
*fimH* (Figure 1E)	63.0	95.9	44.0	49.6
*ompR* (Figure 1D)	56.0	74.5	43.1	88.7
*bolA* (Figure 1H)	48.3	77.6	57.1	78.9
*luxS* (Figure 1G)	59.0	60.9	52.6	74.3

### Biofilm production in *csgD* knockdown EcN and K12 cells

**Figure 2:**
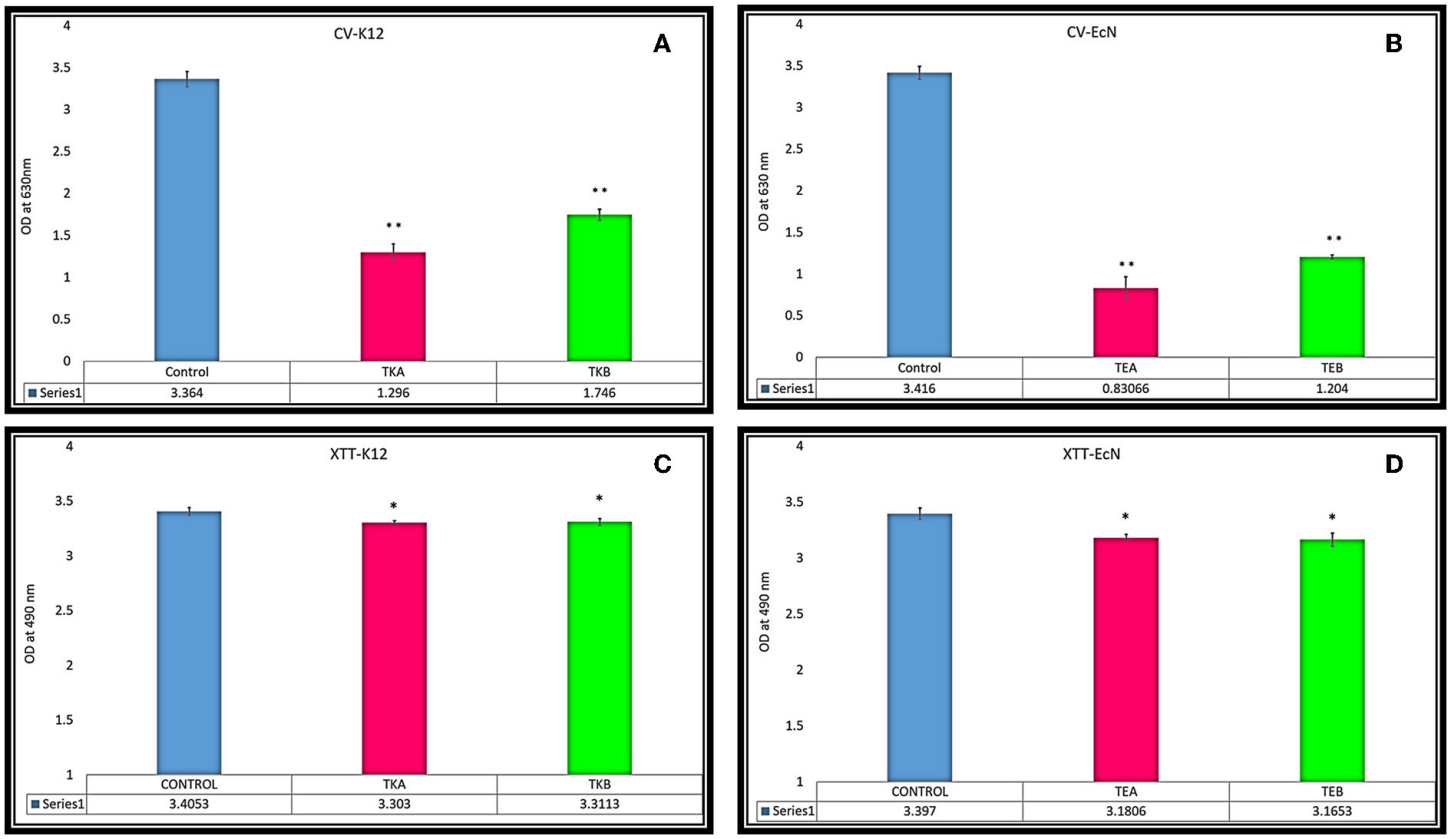
**(A,B)** Biofilm reduction quantification was done through CV assay in *csgD*-KD cells. **(A)** TKA showed 61.47% and TKB 48.09% biofilm reduction, respectively, compared to the control cells. **(B)** The EcN *csgD*-KD mutants TEA and TEB showed 75.6% and 64.7% biofilm reduction. **(C,D)** XTT reduction assay for cell viability in *csgD csgD*-KD cells. **(C)** Cell viability of *csgD*-KD cells of K12 (TKA and TKB) **(D)** Cell viability of *csgD*-KD cells of EcN (TEA and TEB). The data shown here represent an average value of triplicate experiments ± S (*P < 0.05, *t*-test, two-sided), (**P < 0.005, *t*-test, two-sided).

Crystal violet (CV) assay was performed to assess the biofilm production in both EcN and K12 cells. In both K12 *csgD*-KD cells, TKA and TKB showed 61.47 and 48.09% biofilm reduction, respectively, compared to the control cells. While in the case of EcN KD mutants, TEA and TEB showed 75.6 and 64.7% reduction in biofilm formation, respectively (Figures 2A,B).

### Cell viability of *csgD*-KD cells

The XTT assay of *csgD-*KD K12 (TKA and TKB) and EcN (TEA and TEB) showed that *csgD-*KD mutation has no considerable effect on cell viability. TKA and TKB showed the same viability of about 97% as compared to the control cells, while the TEA and TEB showed cell viability of 93.6 and 93.1%, respectively (Figures 2C,D).

### Thioflavin and Congo red fluorescence assay for amyloid production

**Figure 3:**
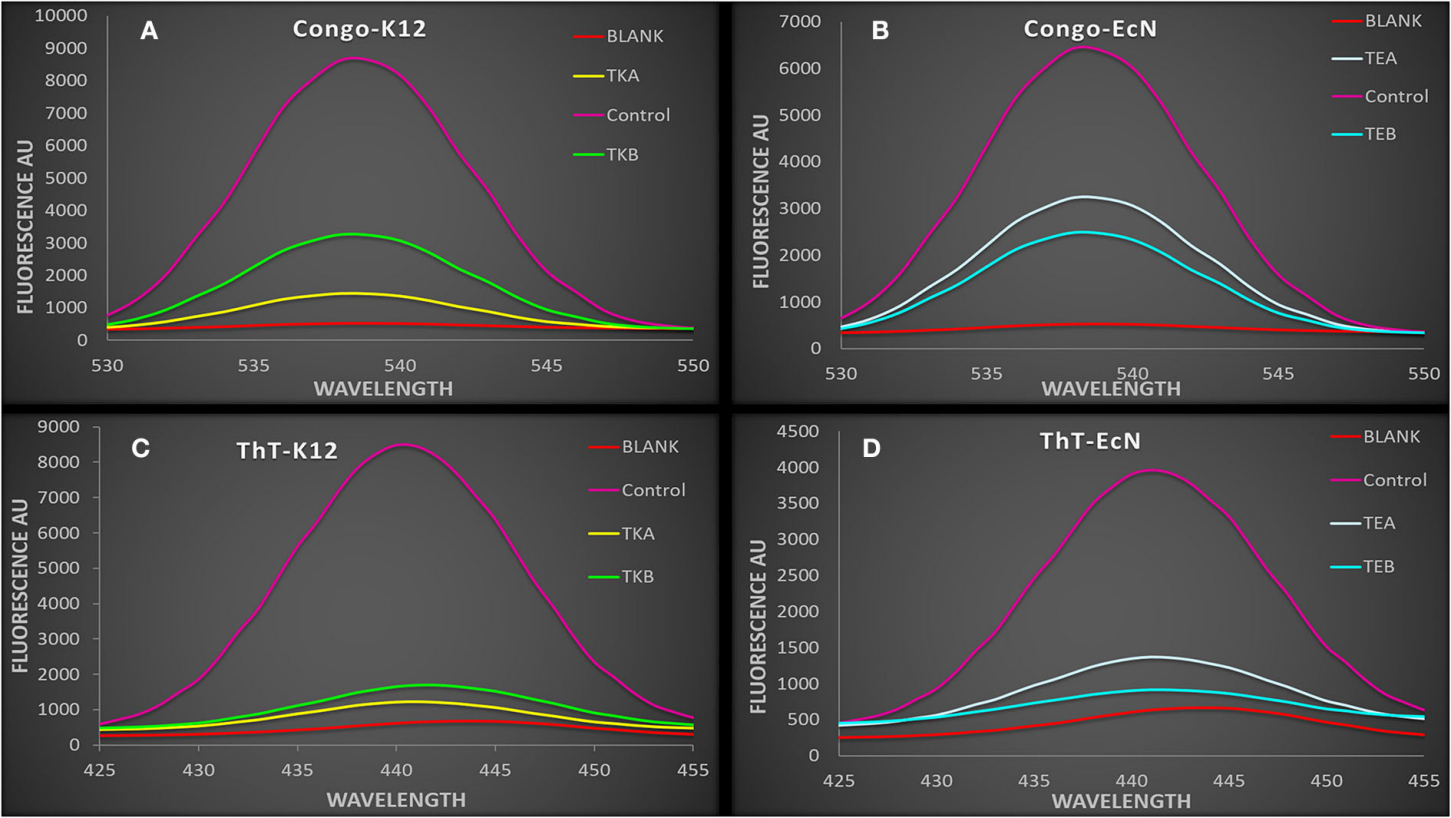
**(A,B)** The spectra showing Congo red fluorescence assay of *csgD*-KD cells. The control cell showed a higher fluorescence intensity as compared to the *csgD*-KD cells **(A)** Congo red fluorescence assay of *csgD*-KD cells of K12 (TKA and TKB) **(B)** Congo red fluorescence assay of *csgD*-KD cells of EcN (TEA and TEB). **(C,D)** The spectra are showing ThT (thioflavin) fluorescence assay in *csgD*-KD cells. The decline in fluorescence spectra intensity suggests lower curli amyloid production in *csgD*-KD cells, **(C)** ThT fluorescence assay of TKA and TKB, **(D)** ThT fluorescence assay of TEA and TEB.

The *csgD-*KD K12 cells (TKA and TKB) showed a reduced ThT and Congo red fluorescence intensity as compared to their controls biofilm cells. Similarly, EcN cells with csgD mutation (TEA and TEB) also showed a decreased fluorescence intensity compared to the control EcN biofilm cells having pdCas9 and sgRNA plasmid with no aTC induction (Figure 3). This decrease in the ThT and Congo red fluorescence intensity in *csgD*-KD indicates a reduction in the curli amyloid fiber production.

### Curli production on Congo red agar plate

**Figure 4:**
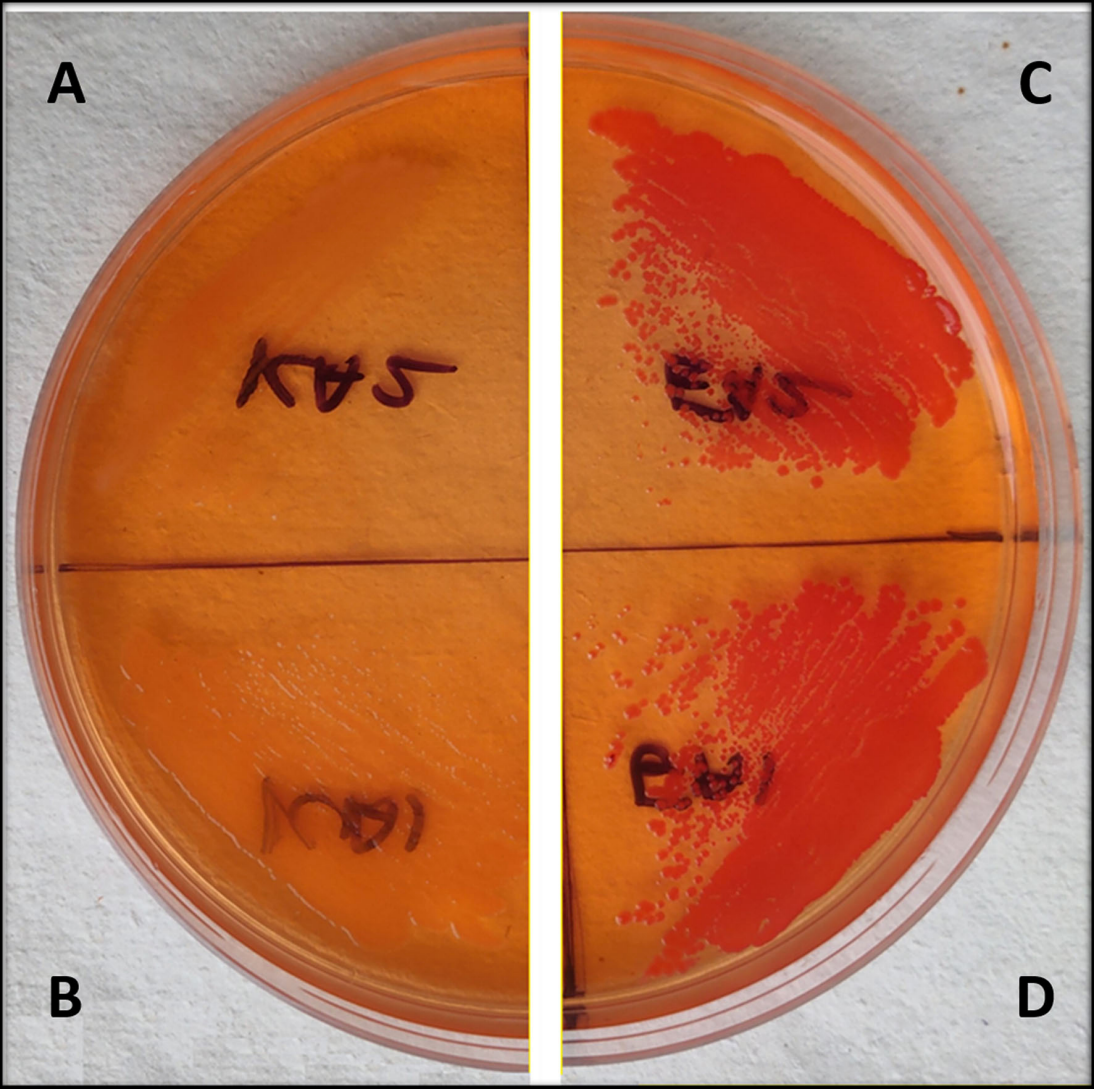
**(A–D)** Congo red agar plate assay of EcN and K12 cells with csgD knockdown. **(A,B)** show *csgD*-KD cells of EcN and K12, respectively, while **(C,D)** show control cells of EcN and K12. The Congo red agar plates show a yellowish-white colony in mutated cells, suggesting a decrease in the curli production.

Congo red plates showed a decrease in the curli production in *csgD* gene knockdown strains as compared to the control cells, which is having no aTc induction. The *csgD*-KD K12 and EcN cells appeared yellowish-white in color, while the wild-type K12 and EcN cells seemed to be dark red colored. The dark red color indicates a higher curli production in control cells as compared to the *csgD*-KD (Figures 4A–D).

### Microscopic studies

**Figure 5:**
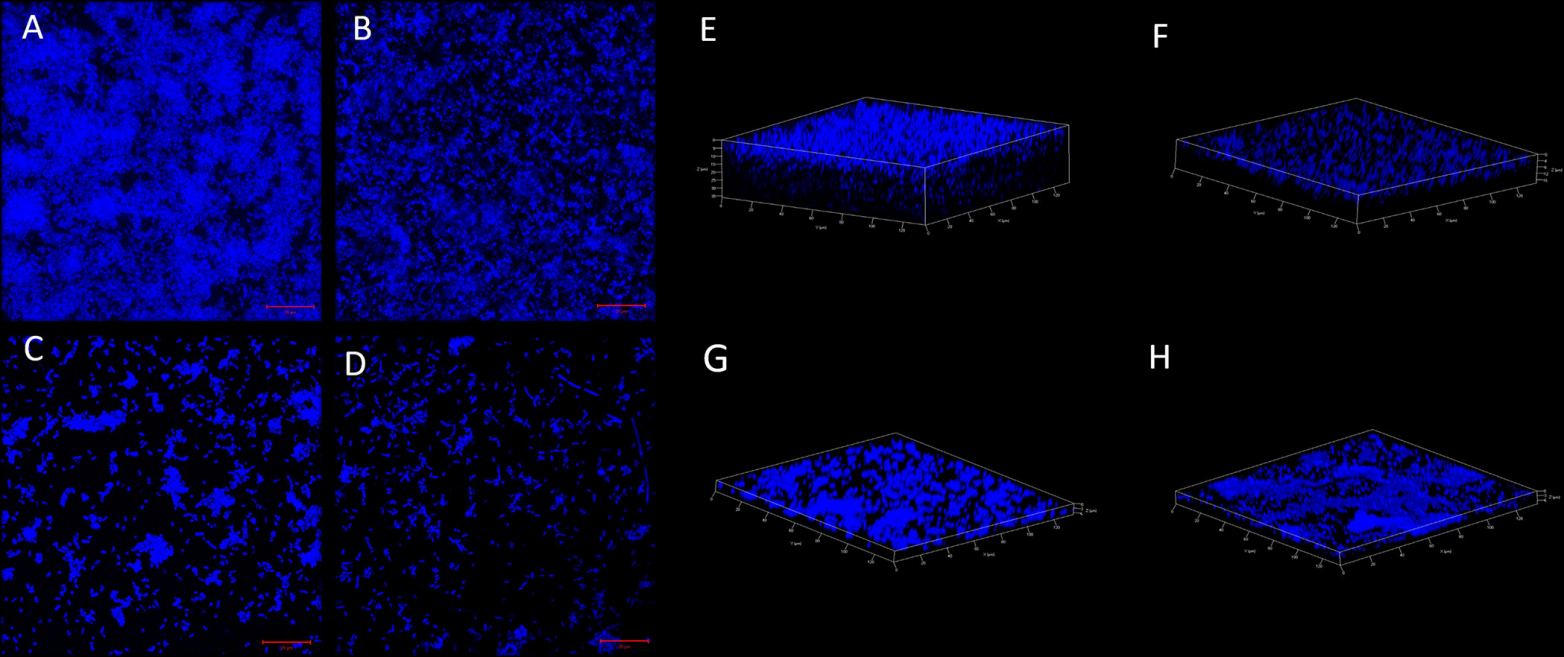
**(A–D)** Biofilm cell adherence assay through CSLM. **(A,B)** show control EcN and K12 cells while the **(C,D)** show *csgD*-KD cells of EcN and K12. The control cells **(A,B**) robust biofilm and aggregated cells have high EPS content, while the *csgD*-KD cells show a reduction in the EPS contents, as well, as the cells seem to be dispersed. The images were taken on a 20-μm scale. **(E–H)** 3D confocal images are showing biofilm thickness. **(E,F)** show control EcN and K12 cells, while **G,H** show *csgD*-KD cells of EcN and K12. The *csgD*-KD cells show a reduction in the biofilm thickness as compared to the control cells. In this figure and 6 images were taken at a 20-μm scale and on 63X magnification.

Confocal microscopy (CLSM) was performed to visualize the biofilm production and adherence property of the *csgD*-KD K12 and EcN cells. A decrease in fluorescence suggests the loss of EPS DNA content and the loss of biofilm structure in the *csgD*-KD compared to their respective control biofilm cells. The CLSM observation also dispersed cells indicating cell aggregation property reduction and also reduction in the thickness of the biofilm. A decrease in fluorescence cell aggregation and adherence ultimately suggests the loss of EPS content and the loss of biofilm structure in the *csgD*-KD compared to their respective control biofilm cells (Figure 5).

**Figure 6:**
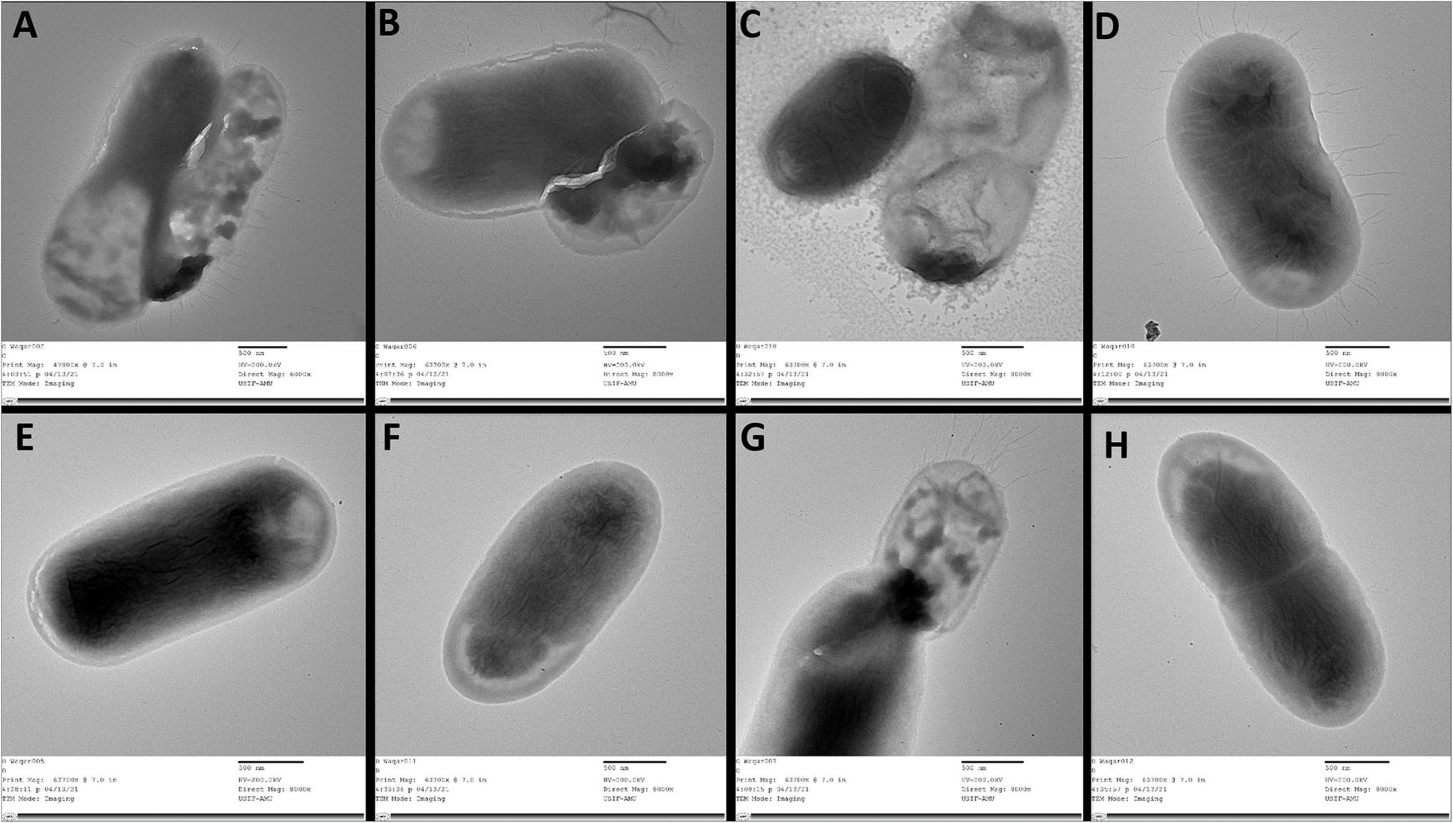
TEM images of *csgD-*KD cells of EcN and K12. **(A–D)** shows control of TEA, TKA, TEB, and TKB, respectively, while **(E–H)** represents the *csgD*-KD cells of TEA, TKA, TEB, and TKB. The control cells show a large number of fimbriae, curli amyloid fibers, and other cell surface extremities, while the *csgD*-KD cells show no fimbriae on their cell surface. All images were taken on a 500-nm scale.

TEM study was performed to visualize the changes on the cell surface of the *csgD*-KD cells (Figure 6). The TEM results are in coherence with the results mentioned above, and *csgD*-KD cells show a remarkable reduction in the cell surface extremities.

**Figure 7:**
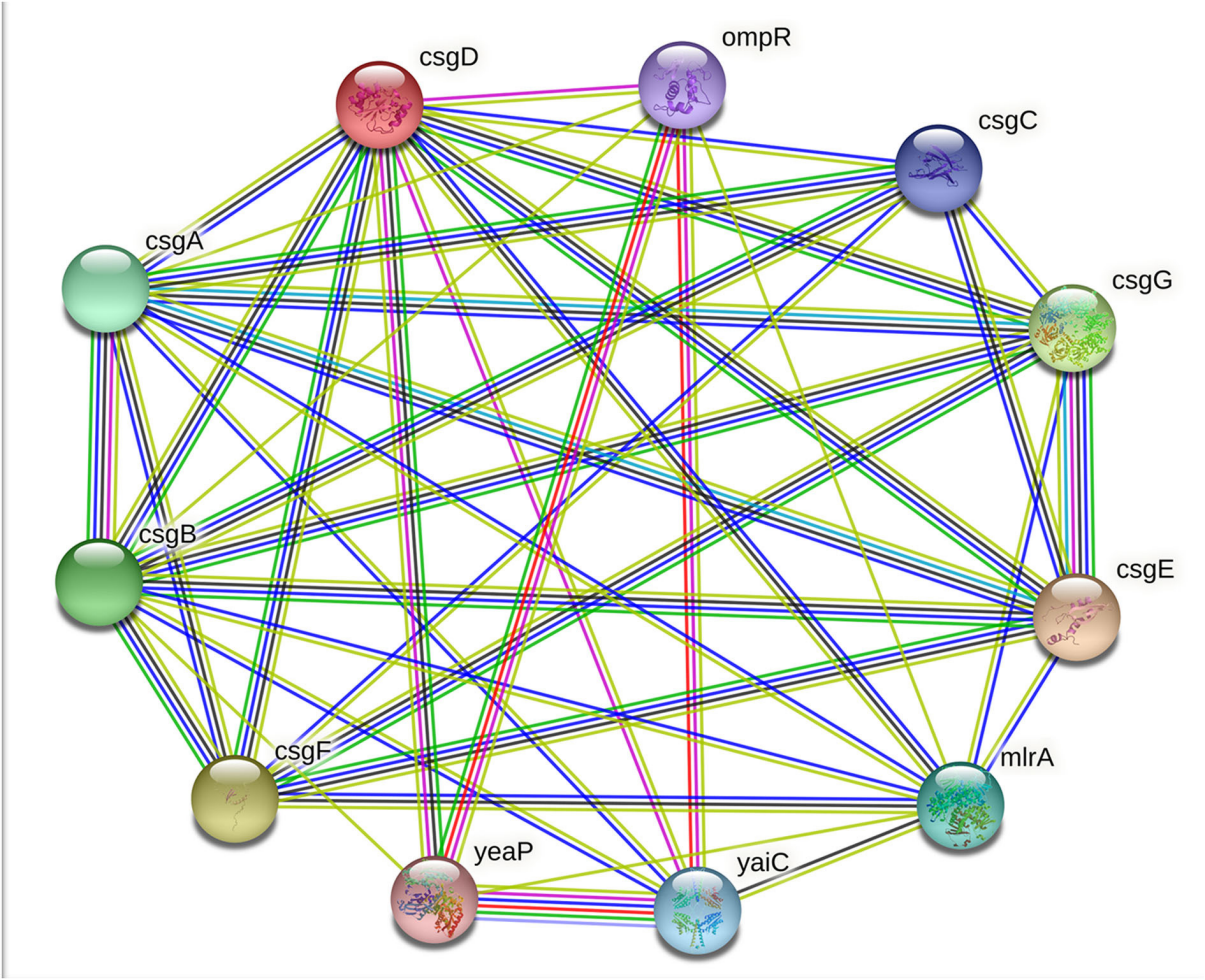
String image showing CsgD and its interaction with CsgA, CsgB, OmpR, and other proteins.

The extracellular polymeric substances (EPS) of bacterial biofilms are composed of several exopolysaccharides and secreted proteins; some of these proteins can form amyloid fibers, RNA, extracellular DNA (eDNA), and lipid molecules. Unlike the toxic amyloids, for example, the amyloids associated with neurodegenerative diseases in humans, curli amyloid fibers, are functional amyloids. In addition to the enteric bacteria, amyloid fibers or amyloid-like protein fibers are also reported in several other Gram-negative and Gram-positive bacteria biofilm matrices. For example, TasA fibers in *Bacillus subtilis;* phenol-soluble modulins in *S. aureus;* Fab fibers in many *Pseudomonas* species; amyloidogenic protein pore-forming bacteriocin microcin *E492* (MccE492) in *Klebsiella pneumoniae*; secreted SMU_63c protein and fragments of cell-surface-localized adhesins P1 and WapA in *S. mutans* (24, 25). In *E. coli*, these amyloid fibers are formed from polymerisation of the amyloidogenic protein subunit of curli fibers CsgA in a membrane-anchored nucleator protein CsgB subunit induced process. Many other proteins and transcriptional regulators are also required for amyloid production, including molecular channels that allow the curli subunits secretion and molecular chaperones, which prohibits uncontrolled aggregation (26). The principal subunit of curli amyloid fibers CsgA is encoded by the *csgA* gene under the regulation of the master regulator of adhesive curli fimbriae expression CsgD. The *csgD* gene encodes the CsgD protein, and it is classified under the LuxR response regulatory family with a conserved Helix-Turn-Helix (HTH) DNA-binding motif (27). As mentioned above, the CsgD protein activates the *csgBAC* operon transcription. Besides the CsgD-led *csgBAC* activation, the curli production is controlled by a complex regulatory mechanism involving both operons: c*sgBAC* operon to encode curli structural subunits and *csgDEFG* operon to encode the CsgD transcription regulator and the CsgEFG curli-specific transport system (Figure 7) (28).

**Figure 8:**
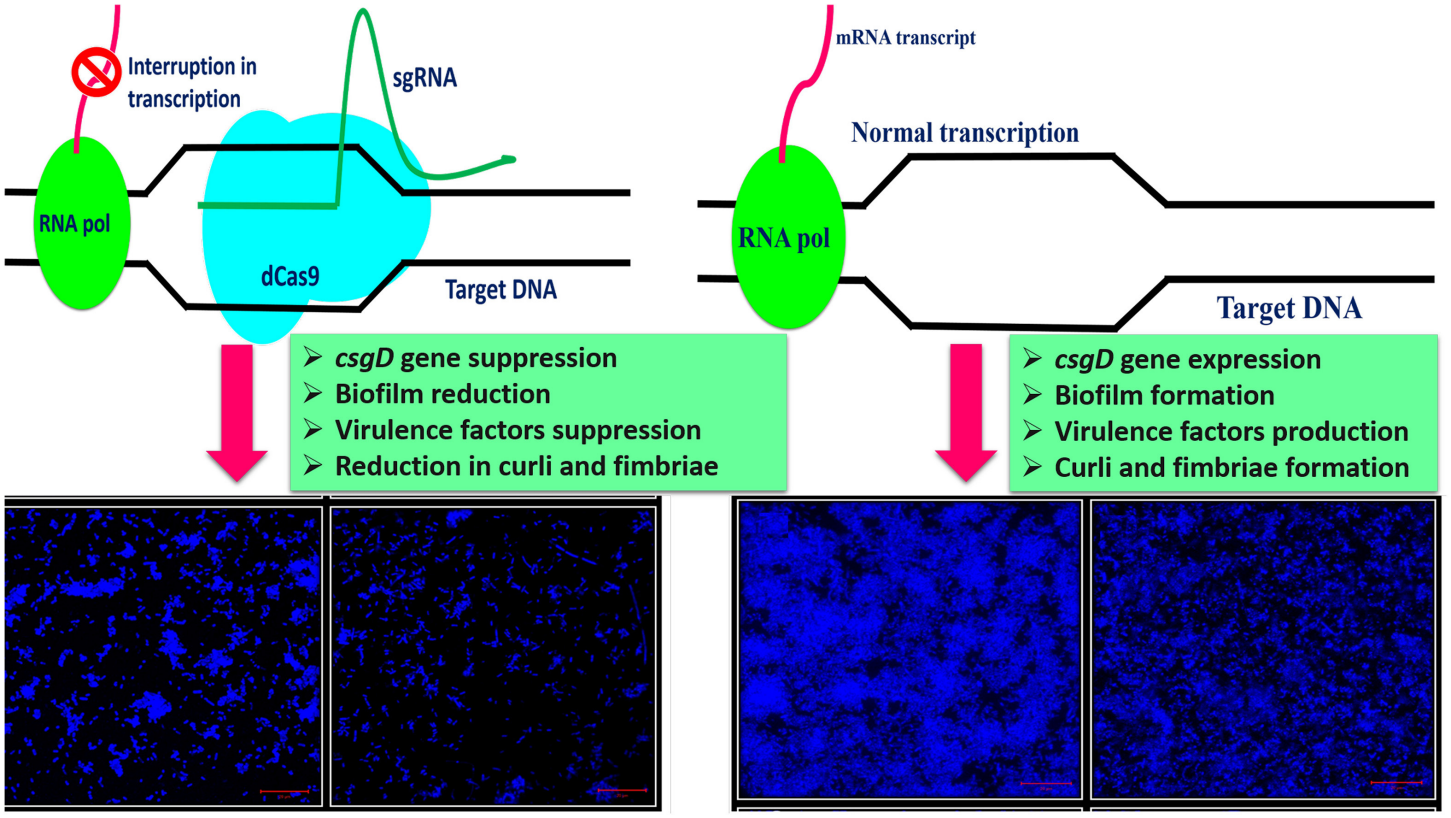
A comprehensive overview of the study.

This study aimed to observe the effects of *csgD* gene mutation in two different *E. coli* strains viz. *E. coli* Nissle 1917 and K12 and its impact on curli amyloid production and other genes critical to the biofilm formation, cell adhesion, and virulence factors production (Figure 8). CRISPRi-mediated *csgD*-KD cells of *E. coli* Nissle 1917 and K12 were generated to set the foundation of our study.

The RT-PCR data showed more than 35% of *csgD* gene suppression in both *csgD*-KD of EcN (TEA and TEB) and K12 cells (TKA and TKB). TEA *csgD*-KD of EcN shows 65.8% suppression and TKA *csgD*-KD of K12 cells show 63.8% suppression, while in TEB *csgD*-KD of EcN, 35.4% gene suppression was observed, and TKB *csgD*-KD of K12 cells showed 41% suppression of *csgD* (Figure 1A). Since these two mutants are located at different positions in the *csgD* gene, it may be because the gene expression data shows the variation in the gene expression in the same cell.

The transition of *Escherichia coli* cells from the planktonic solitary growth phase to the biofilm phase led to several changes in the cell's regulatory mechanism, such as genes of flagellum formation turned off and CsgD activates the synthesis of curli fimbriae and other extracellular polysaccharides formation for cell-cell adhesion (29). The capability of adhesion on host cells in *E. coli* has a most critical role in the colonization of any site, and adhesins mediate cell adhesion in *E. coli*. Adhesins specifically recognize receptors, and they are either carried by fimbriae/pili or directly exposed to the bacterial cell surface. A majority of the adhesins are encoded by the pathogenicity islands (PAIs) (30). Fimbriae (pili) are filamentous structures that help bacterial cell adherence to the cell receptors of the host. More than 80% of *E. coli* strains and other Enterobacteriales possess type 1 fimbriae, and they are known to play a significant role in ExPEC virulence. The expression of type 1 fimbriae is regulated by PAIs and is critical for bladder colonization by UPEC (29, 31). Type 1 fimbriae are encoded by fimbriae (*fim*) gene, *fimAICDFGH*. The *fimA* gene encodes FimA, the major protein subunit of fimbriae, while the *fimH* gene encodes the FimH subunit. The FimH is the main adhesin protein of type 1 fimbria, located at the tip of the fimbriae. It binds specifically to d-mannosylated receptors such as uroplakins and is present in an abundant amount on the surface of bladder cells, enterocytes, endothelial cells of brain capillaries, and other mannosides (Co6) containing surfaces of host cells. At least three regulatory proteins (Lrp, IHF, and H-NS) are directly involved in the *fim* genes regulation, and many other environmental factors are also affecting its regulation, including temperature variation, pH change, osmolarity, and level of oxygen (30, 31).

To observe the effect of *csgD* gene suppression on other genes critical to biofilm formation, we have assessed the expressions *csgA, csgB, fimA, fimH, ompR, luxS*, and *bolA* genes in *csgD*-KD. The qRT-PCR data showed the downregulation of all these genes in the *csgD*-KD of both EcN and K12 cells (Table 1). As mentioned above, the *csgA* and *csgB* genes encode the structural components as CsgA and CsgB protein subunit of curli fibers, and CsgD activates the expression of the *csgBAC* operon. The RT-PCR data revealed both *csgA* and *csgB* gene downregulation in both *csgD*-KD of EcN and K12 cells (Figures 1B,C). The *fimA* gene encodes the major fimbriae subunit FimA and *fimH* encodes the adhesion portion located at the tip of fimbriae, FimH subunit. The *ompR* gene is involved in EnvZ-OmpR two-component systems (TCS) of the bacterial signal transduction system, and it has been reported that *ompR* regulates more than 125 genes in *E. coli* (32). The OmpR/EnvZ system is essential for *csgD gene* expression, and it responds to changes in osmolarity (33). Our gene expression data showed downregulation of *ompR* gene expression in *csgD*-KD of EcN and K12 cells (Figure 1D). The exact mechanism of the *csgD* gene knockdown, which leads to the downregulation of the *ompR* gene, is yet unknown. The *luxS* gene plays a vital role in the bacterial quorum-sensing mechanism, a cell-cell communication system, by involving in the biosynthesis (QS) of signal molecule autoinducer-2 (AI2). After reaching a threshold cell density, signal molecule AI2 promotes biofilm formation by activating transcription factors. It was also reported that *fimH* deleted strain shows a downregulation in the *luxS* gene expression (34). Our qRT-PCR data of *csgD*-KD cells show the downregulation of both *fimH* and *luxS* genes in all *csgD*-KD cells, that is, *csgD* gene downregulated cells of EcN and K12 (Figures 1E,G). The *csgD*-KD also shows the downregulation of the *bolA* gene (Figure 1H). The *bolA* gene is a transcriptional regulator for cell shape in *E. coli*, and it is also known to play a vital role in biofilm development. The *bolA* promotes biofilm formation by regulating the proteins related to biofilm development (35).

It was mentioned above that *E. coli* Nissle 1917 expresses several virulence factors. It shares many fitness factors UPEC CFT073, including iron acquisition mechanisms, production of the capsule, antimicrobial agents production, and adhesins production (19, 20). These virulence factors of the Nissle strain make it pathogenic. Nissle 1917 also produces genotoxin colibactin, which has pro-carcinogenic properties (36, 37).

We have performed a crystal violet (CV) biofilm quantification assay to observe the reduction in biofilm formation in these *csgD*-KD cells. All four *csgD*-KD cells showed more than 45% reduction in biofilm formation. Similar to the qRT-PCR results, “*csgD*-KD A mutants” show higher *csgD* gene suppression than “*csgD*-KD B mutants”, and in CV assay “*csgD*-KD A mutants” of both cells (TEA and TKA) showed more reduction in biofilm formation than the “*csgD*-KD B mutants” (TEB and TKB) as compared to the control biofilm cells (Figures 2A, 3B). We have also performed the confocal microscopic study (CLSM) to validate biofilm and cell adhesion reduction further. The confocal images show that *csgD*-KD cells showed a decrease in cell adherence and cell aggregate formation. The dispersed cells observed in CLSM indicate a reduction in biofilm formation compared to the control (Figures 5A–D). These *csgD*-KD cells also showed a decrease in the biofilm thickness compared to the control cells (Figures 5E–H).

Like all amyloids, the curli fiber amyloids or functional amyloids also bind with amyloid binding dyes such as Congo red (CR) and Thioflavin T (ThT) (38). To check the curli amyloid formation in *csgD*-KD cells, we have performed ThT and Congo red fluorescence assay. The fluorescence spectra of *csgD-*KD cells of K12 (TKA and TKB) and EcN (TEA and TEB) showed a remarkable decrease in ThT and Congo red binding as compared to the control strain, which was grown in the absence of aTc inducer (Figures 3A–D). Further, we also have performed the Congo red agar plate assay to check the curli production. The *csgD*-KD cells of K12 and EcN appeared as whitish-yellow, indicating low curli fimbriae production. In contrast, the control cells appeared as dark red on the Congo red agar plates, which means the higher curli production in control cells (Figures 4A–D). The ThT and Congo red fluorescence assay results, as well as Congo red agar plate assay, suggest a reduction in the curli amyloid production in the *csgD-*KD cells of both K12 and EcN.

The transmission electron microscopic (TEM) study was performed to visualize the curli fimbriae and other cell surface structures in biofilm cells of *csgD*-KD cells of both K12 and EcN cells. The TEM images showed very few curli, fimbriae, and other extracellular extremities on the cell surface of *csgD*-KD cells of K12 (TKA and TKB) and EcN (TEA and TEB). The control cells appeared very robust cells with an abundance of curli, fimbriae, and other cell surface extremities on its cell surface. The TEM results further validated the ThT and Congo red fluorescence assay as well as Congo red agar plate assay showing a decrease in curli amyloid production (Figure 6).

XTT reduction assay was performed to see whether CRISPRi-mediated knockdown mutation of csgD has any effect on cell viability. The XTT [2, 3-bis (2-methoxy-4-nitro-5-sulfophenyl)-2H-tetrazolium-5-carboxanilide] reduction assay is a standard assay to assess the metabolic activity of the biofilm cell (39). The XTT reduction assay revealed that *csgD-*KD cells of TKA, TKB, TEA, and TEB showed 97, 97.2, 93.6, and 93.1% cell viability, respectively (Figures 2C,D). The XTT reduction assay data suggest that CRISPRi-mediated knockdown of the *csgD* gene is having no significant effect on the viability of the cells.

## Conclusion

*E. coli* Nissile 1917 strain is being used as a probiotic for many decades. We have explored the effects of *csgD*–KD mutation on curli amyloid production and on different genes involved in biofilm formation, as well as the expression of virulence factors in EcN strain, and the obtained results were compared with the nonpathogenic *E. coli* K12 strain. Hence, *csgD* gene inhibition through the CRISPRi technique may be used as an approach for the improvement of the probiotic *E. coli* Nissle 1917 strain and other probiotic strains of medicinal importance.

## Data availability statement

The original contributions presented in the study are included in the article/Supplementary material, further inquiries can be directed to the corresponding author.

## Ethics statement

The study was approved by the Institutional Biosafety Committee.

## Author contributions

MA and AK designed the problem. MA performed all experiments, analyzed data, and wrote the first draft of manuscript. AK guided and supported the study, analyzed data, and edited the draft manuscript. All authors contributed to the article and approved the submitted version.

## Funding

This study was financially supported by internal funds of the Interdisciplinary Biotechnology Unit, AMU, Aligarh, India, and DBT Grant No. BT/PR40148/BTIS/137/20/2021.

## Conflict of interest

The authors declare that the research was conducted in the absence of any commercial or financial relationships that could be construed as a potential conflict of interest.

## Publisher's note

All claims expressed in this article are solely those of the authors and do not necessarily represent those of their affiliated organizations, or those of the publisher, the editors and the reviewers. Any product that may be evaluated in this article, or claim that may be made by its manufacturer, is not guaranteed or endorsed by the publisher.

## References

[1] Surgers L Boyd A Girard PM Arlet G Decré D. Biofilm formation by ESBL-producing strains of *Escherichia coli* and *Klebsiella pneumoniae*. Int J Med Microbiol. (2019) 309:13–18. 10.1016/j.ijmm.2018.10.008 30385204

[2] Flament-Simon SC Duprilot M Mayer N García V Alonso MP Blanco J. Association between kinetics of early biofilm formation and clonal lineage in *Escherichia coli*. Front Microbiol. (2019) 10:1183. 10.3389/fmicb.2019.01183 31214138 PMC6555128

[3] Shaler CR Elhenawy W Coombes BK. The unique lifestyle of crohn's disease-associated adherent-invasive *Escherichia coli*. J Mol Biol. (2019) 431:2970–2981. 10.1016/j.jmb.2019.04.023 31029703

[4] Fleckenstein JM Kuhlmann FM. Enterotoxigenic *Escherichia coli* infections. Curr Infect Dis Rep. (2019) 21:9. 10.1007/s11908-019-0665-x 30830466 PMC6508951

[5] Reitzer L Zimmern P. Rapid growth and metabolism of uropathogenic escherichia coli in relation to urine composition. Clin Microbiol Rev. (2020) 33:1–20. 10.1128/CMR.00101-19 31619395 PMC6927312

[6] Riley LW. Extraintestinal foodborne pathogens. Annu Rev Food Sci Technol. (2020) 11:275–94. 10.1146/annurev-food-032519-051618 32004092

[7] Deng Z Luo XM Liu J Wang H. Quorum sensing, biofilm, and intestinal mucosal barrier: involvement the role of probiotic. Front Cell Infect Microbiol. (2020) 10:538077. 10.3389/fcimb.2020.538077 33102249 PMC7546212

[8] Fang K Jin X Hong SH. Probiotic Escherichia coli inhibits biofilm formation of pathogenic *E. coli* via extracellular activity of DegP. Sci Rep. (2018) 8:4939. 10.1038/s41598-018-23180-1 29563542 PMC5862908

[9] Bhoite S van Gerven N Chapman MR Remaut H. Curli biogenesis: bacterial amyloid assembly by the type VIII secretion pathway. Protein Secret Bact. (2019) 8:163–171. 10.1128/9781683670285.ch14 30892177 PMC6428212

[10] Sewell L Stylianou F Xu Y Taylor J Sefer L Matthews S. NMR insights into the pre-amyloid ensemble and secretion targeting of the curli subunit CsgA. Sci Rep. (2020) 10:7896. 10.1038/s41598-020-64135-9 32398666 PMC7217966

[11] Miller AL Pasternak JA Medeiros NJ Nicastro LK Tursi SA Hansen EG. In vivo synthesis of bacterial amyloid curli contributes to joint inflammation during *S. typhimurium* infection. PLoS Pathog. (2020) 16:e1008591. 10.1371/journal.ppat.1008591 32645118 PMC7347093

[12] Levkovich SA Gazit E Laor Bar-Yosef D. Two decades of studying functional amyloids in microorganisms. Trends Microbiol. (2020) 29:251–265. 10.1016/j.tim.2020.09.005 33041179

[13] Jain N Chapman MR. Bacterial functional amyloids: order from disorder. Biochim Biophys Acta Proteins Proteomics. (2019) 1867:954–60. 10.1016/j.bbapap.2019.05.010 31195143 PMC6661199

[14] Ogasawara H Ishizuka T Yamaji K Kato Y Shimada T Ishihama A. Regulatory role of pyruvate-sensing BtsSR in biofilm formation by *Escherichia coli* K-12. FEMS Microbiol Lett. (2020) 366:fnz251. 10.1093/femsle/fnz251 31834370

[15] Nhu NTK Phan MD Peters KM Lo AW Forde BM Chong TM. Discovery of new genes involved in curli production by a uropathogenic *Escherichia coli* strain from the highly virulent O45:K1:H7 lineage. MBio. (2018) 9:e01462–18. 10.1128/mBio.01462-18 30131362 PMC6106082

[16] Miller AL Bessho S Grando K Tükel Ç. Microbiome or infections: amyloid-containing biofilms as a trigger for complex human diseases. Front Immunol. (2021) 12:638867. 10.3389/fimmu.2021.638867 33717189 PMC7952436

[17] Seo DO Holtzman DM. Gut microbiota: From the forgotten organ to a potential key player in the pathology of Alzheimer's disease. J Gerontol Ser A Biol Sci Med Sci. (2020) 75:1232–41. 10.1093/gerona/glz262 31738402 PMC7302187

[18] Sampson TR Challis C Jain N Moiseyenko A Ladinsky MS Shastri GG. A gut bacterial amyloid promotes a-synuclein aggregation and motor impairment in mice. Elife. (2020) 9:e53111. 10.7554/eLife.53111 32043464 PMC7012599

[19] Pradhan S Weiss AA. Probiotic properties of *Escherichia coli* nissle in human intestinal organoids. MBio. (2020) 11:e01470–20. 10.1128/mBio.01470-20 32636253 PMC7343996

[20] Hancock V Dahl M Klemm P. Probiotic *Escherichia coli* strain Nissle 1917 outcompetes intestinal pathogens during biofilm formation. J Med Microbiol. (2010) 59:392–9. 10.1099/jmm.0.008672-0 20110388

[21] Larson MH Gilbert LA Wang X Lim WA Weissman JS Qi LS. CRISPR interference (CRISPRi) for sequence-specific control of gene expression. Nat Protoc. (2013) 8:2180–96. 10.1038/nprot.2013.132 24136345 PMC3922765

[22] Zuberi A Ahmad N Khan AU. CRISPRi induced suppression of fimbriae gene (fimH) of a uropathogenic *Escherichia coli*: an approach to inhibit microbial biofilms. Front Immunol. (2017) 8:1552. 10.3389/fimmu.2017.01552 29181009 PMC5694031

[23] Zuberi A Misba L Khan AU. CRISPR interference (CRISPRi) inhibition of luxS gene expression in *E. coli*: An approach to inhibit biofilm. Front Cell Infect Microbiol. (2017) 7:214. 10.3389/fcimb.2017.00214 28603699 PMC5445563

[24] Azam MW Zuberi A Khan AU Khan AU. BolA gene involved in curli amyloids and fimbriae production in *E. coli*: exploring pathways to inhibit biofilm and amyloid formation. J Biol Res. (2020) 27:10. 10.1186/s40709-020-00120-7 32566535 PMC7301969

[25] Pruteanu M Hernández Lobato JI Stach T Hengge R. Common plant flavonoids prevent the assembly of amyloid curli fibres and can interfere with bacterial biofilm formation. Environ Microbiol. (2020) 22:5280–99. 10.1111/1462-2920.15216 32869465

[26] Marcoleta A Wien F Arluison V Lagos R Giraldo R. Bacterial amyloids. In: eLS. Chichester: John Wiley & Sons, Ltd. (2019). p. 1–9. 10.1002/9780470015902.a0028401

[27] Wen Y Ouyang Z Devreese B He W Shao Y Lu W. Crystal structure of master biofilm regulator CsgD regulatory domain reveals an atypical receiver domain. Protein Sci. (2017) 26:2073–82. 10.1002/pro.3245 28758290 PMC5606546

[28] Brombacher E Baratto A Dorel C Landini P. Gene expression regulation by the curli activator CsgD protein: modulation of cellulose biosynthesis and control of negative determinants for microbial adhesion. J Bacteriol. (2006) 188:2027–37. 10.1128/JB.188.6.2027-2037.2006 16513732 PMC1428138

[29] Ogasawara H Yamamoto K Ishihama A. Role of the biofilm master regulator CsgD in cross-regulation between biofilm formation and flagellar synthesis. J Bacteriol. (2011) 193:2587–97. 10.1128/JB.01468-10 21421764 PMC3133154

[30] Bessaiah H Pokharel P Habouria H Houle S Dozois CM. YqhG contributes to oxidative stress resistance and virulence of uropathogenic *Escherichia coli* and identification of other genes altering expression of type 1 fimbriae. Front Cell Infect Microbiol. (2019) 9:312. 10.3389/fcimb.2019.00312 31555608 PMC6727828

[31] Desvaux M Dalmasso G Beyrouthy R Barnich N Delmas J Bonnet R. Pathogenicity factors of genomic islands in intestinal and extraintestinal *Escherichia coli*. Front Microbiol. (2020) 11:2065. 10.3389/fmicb.2020.02065 33101219 PMC7545054

[32] Rentschler AE Lovrich SD Fitton R Enos-Berlage J Schwan WR. OmpR regulation of the uropathogenic escherichia coli fimB gene in an acidic/high osmolality environment. Microbiol. (2013) 159:316–27. 10.1099/mic.0.059386-0 23175504 PMC3709560

[33] Andreassen PR Pettersen JS Szczerba M Valentin-Hansen P Møller-Jensen J Jørgensen MG. SRNA-dependent control of curli biosynthesis in *Escherichia coli*: McaS directs endonucleolytic cleavage of csgD mRNA. Nucleic Acids Res. (2018) 46:6746–60. 10.1093/nar/gky479 29905843 PMC6061853

[34] Chen T Liu N Ren P Xi X Yang L Sun W. Efficient biofilm-based fermentation strategies for L-threonine production by *Escherichia coli*. Front Microbiol. (2019) 10:1773. 10.3389/fmicb.2019.01773 31428070 PMC6688125

[35] Silva A V. Edel M Gescher J Paquete CM. Exploring the effects of bolA in biofilm formation and current generation by *Shewanella oneidensis* MR-1. Front Microbiol. (2020) 11:815. 10.3389/fmicb.2020.00815 32457717 PMC7225295

[36] Bian X Plaza A Zhang Y Müller R. Two more pieces of the colibactin genotoxin puzzle from *Escherichia coli* show incorporation of an unusual 1-aminocyclopropanecarboxylic acid moiety. Chem Sci. (2015) 6:3154–60. 10.1039/C5SC00101C 28706687 PMC5490422

[37] Lopès A Billard E Casse AH Villéger R Veziant J Roche G. Colibactin-positive *Escherichia coli* induce a procarcinogenic immune environment leading to immunotherapy resistance in colorectal cancer. Int J Cancer. (2020) 146:3147–59. 10.1002/ijc.32920 32037530

[38] Evans ML Gichana E Zhou Y Chapman MR. Bacterial amyloids. Methods Mol Biol. (2018) 1779:267–88. 10.1007/978-1-4939-7816-8_17 29886539 PMC6447394

[39] Lu C Liu H Shangguan W Chen S Zhong Q. Antibiofilm activities of the cinnamon extract against *Vibrio parahaemolyticus* and *Escherichia coli*. Arch Microbiol. (2020) 203:125–35. 10.1007/s00203-020-02008-5 32772125

