# Supplementary material for: CRISPRi-mediated suppression of E. coli Nissle 1917 virulence factors: A strategy for creating an engineered probiotic using csgD gene suppression
Source: Front Nutr. 2022 Aug 1;9:938989. doi: 10.3389/fnut.2022.938989 (PMC9376613; doi:10.3389/fnut.2022.938989)
Supplement: Supplementary file 1 [file Data_Sheet_1.pdf]

CRISPRi-mediated suppression of *E. coli* Nissle 1917 virulence factors: A strategy for creating an engineered probiotic using *csgD* gene suppression

Mohd W. Azam, and Asad U Khan*

Medical Microbiology and Molecular Biology Lab., Interdisciplinary Biotechnology Unit, Aligarh Muslim University, Aligarh – 202 002, India.

***Corresponding author:** Prof. Asad U. Khan, Medical Microbiology and Molecular Biology Lab., Interdisciplinary Biotechnology Unit, Aligarh Muslim University, Aligarh – 202 002, UP, India. Ph.: 0091-9837021912, Fax: 0091-571-2721776

Email id: asad.k@rediffmail.com

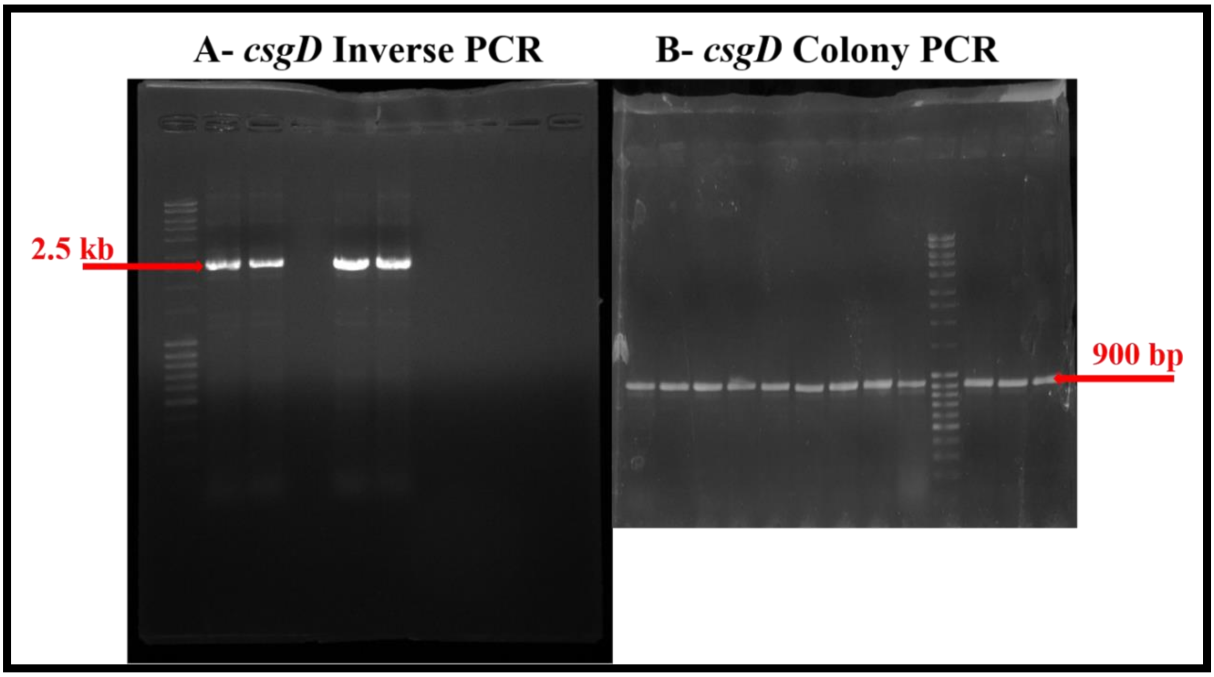

Figure S1: (A) Inverse PCR products are showing 2.5 kb sgRNA plasmid. **(B)** Colony PCR products of the clones with desired sgRNAs.

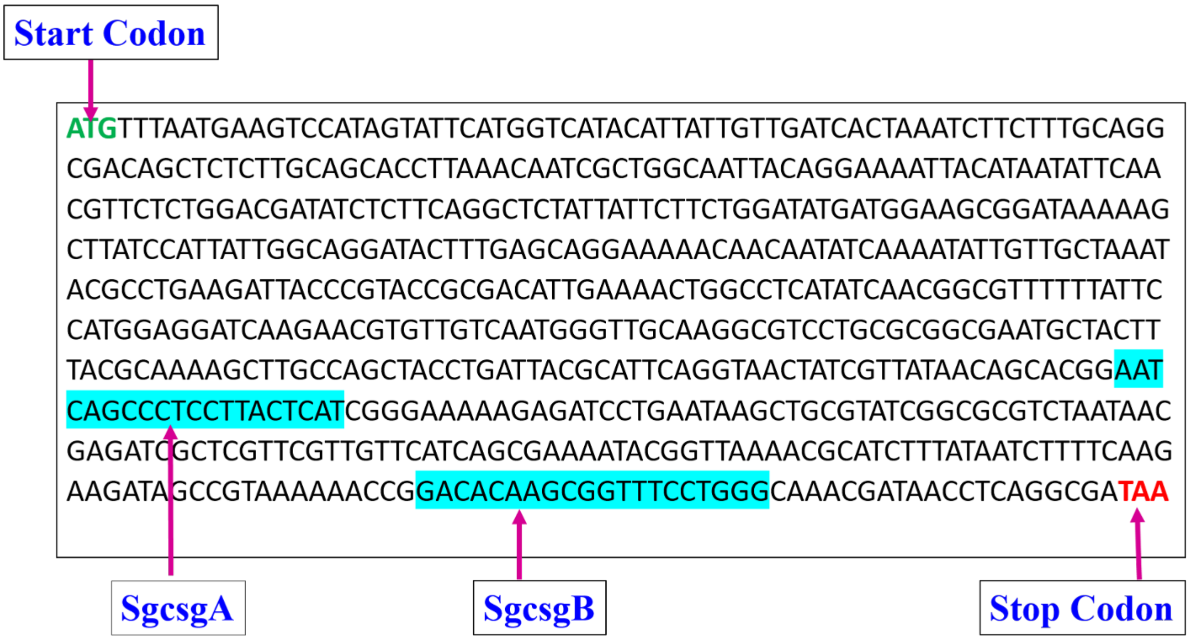

Figure S2: Showing the gene sequences and binding sites of SgcsgA and SgcsgB sgRNA sequences.

Table S1: List of the primers used in this study

Primer name	Sequences
**SgcsgA**	CCCAGGAAACCGCTTGTGTCGTTTTAGAGCTAGAAATAGCAAGTTAAAATAAGGC
**SgcsgB**	AATCAGCCCTCCTTACTCATGTTTTAGAGCTAGAAATAGCAAGTTAAAATAAGGC
**Sgcsg-R**	ACTAGTATTATACCTAGGACTGAGCTAGC
**F-colony**	GGGTTATTGTCTCATGAGCGGATACATATTTG
**R-colony**	CGCGGCCTTTTTACGGTTC
**Lux-RT-F**	GTGTTCGATCTGCGCTTCTG
**Lux-RT-R**	GGATCCCTCTTTCTGGCATCA
**Bol-RT-F**	CCAACCCGTATTCCTCGAAGT
**Bol-RT-R**	GCCGGCTGGGACATTG
**FimA RT-F**	CCTGAATAACGGAACCAATACCA
**FimA RT-R**	GCCCCGGTTGCAAAATAA
**FimH RT-F**	GATGCGGGCAACTCGATT
**FimH RT-R**	CGCCCTGTGCAGGTGAA
**csgA-FW**	GCGGTAATGGTGCAGATGTTG
**csgA-RW**	CGTTGGGTCAGATCGATTGA
**csgB-FW**	CGGCAGGGAGGCTCAAA
**csgB-RW**	CCCGGTTGCTACTACCTTCTTG
**csgD-FW**	CGGAATCAGCCCTCCTTACTC
**csgD-RW**	GCGCCGATACGCAGCTTAT
**ompR-RT-F**	TGCCCGTGGTCGTGAATATT
**ompR-RT-R**	GCGAAATCTGCACGTCGAT
